# A WHO Pathfinder Survey of Dental Caries in 6 and 12-Year Old Transylvanian Children and the Possible Correlation with Their Family Background, Oral-Health Behavior, and the Intake of Sweets

**DOI:** 10.3390/ijerph17114180

**Published:** 2020-06-11

**Authors:** Patricia Ondine Lucaciu, Alexandru Mester, Ioana Constantin, Nora Orban, Lavinia Cosma, Sebastian Candrea, Ruxandra Sava-Rosianu, Anca Stefania Mesaros

**Affiliations:** 1 Department of Oral Health, “Iuliu Hatieganu” University of Medicine and Pharmacy, 400012 Cluj-Napoca, Romania; patricia.lucaciu@umfcluj.ro; 2 Department of Pedodontics, County Hospital Cluj-Napoca, 400000 Cluj-Napoca, Romania; ioanaconstantin11@yahoo.com (I.C.); noraorban@yahoo.com (N.O.); candreasebastian@yahoo.com (S.C.); 3 Department of Pedodontics, “Iuliu Hatieganu” University of Medicine and Pharmacy, 400083 Cluj-Napoca, Romania; dr.laviniacosma@yahoo.com; 4 Department of Preventive Dentistry, Community and Oral Health, “Victor Babes” University of Medicine and Pharmacy, 300195 Timisoara, Romania; savarosianu@yahoo.com; 5 Department of Dental Propaedeutic, “Iuliu Hatieganu” University of Medicine and Pharmacy, 40000 Cluj-Napoca6, Romania; mesaros.anca@umfcluj.ro

**Keywords:** dental health, children, ICDAS, oral health behavior

## Abstract

To our knowledge, recent oral health data in Romania is poor, as no comprehensive oral health surveys have been carried out in the last five years. The present cross-sectional oral health survey aimed to assess the dental health status in 6 and 12-year old children from Transylvania, in correlation with their family background, oral-health behavior, and the intake of sweets. The study was conducted on 290 children from nine schools in the Transylvanian region of Romania. The study consisted of the clinical examination of children, recording of data in an International Cavity Detection and Assessment System (ICDAS) chart, and a questionnaire referring to the child’s parental education, frequency, and motivation of visits to the dentist, dental care habits, and the intake of sweets. Our results indicated that the most prevalent ICDAS scores recorded in 6-year-old children were “0A” (*p* = 0.001464), “03” (*p* = 0.00366), “05” (*p* = 0.005563), “06” for rural areas. Restorations were statistically more prevalent in the urban population (*p* = 0.000076). The ICDAS score for 12-year old children was “03” (*p* = 0.003614) and prevalent in the urban area. The ICDAS score for the rural area was “04” (*p* = 0.0056). Comparing dental health status with family background demonstrated a strong correlation for the group of 6-year-old children, and a lack of correlation for the 12-year-old children. The number of dental visits corelated with the parents’ backgrounds, and was higher in the urban population. Frequent hygiene habits (toothbrushing) were statistically correlated with lower ICDAS scores: “04” (*p* = 0.016482), “05” (*p* = 0.039127), “06” (*p* = 0.010785). Eating habits in both age groups were associated with statistically significant differences of “03”, “04”, “05”, “06”, “0A” in the ICDAS score. The obtained results provided clarification on the dental health situation in Romania and the potential risk factors of caries among the population, and therefore it could be used as a starter point for future studies to investigate, in depth, the effects of various variables on cavities found in Transylvanian schoolchildren.

## 1. Introduction

Tooth decay is considered among the most prevalent diseases in children, affecting about 50% children across the globe [1]. It is a multifactorial, chronic childhood disease, and it is the result of multiple etiological factors such as dietary habits, oral microorganisms that ferment sugars, and host susceptibility. These factors have to coexist for dental caries to initiate and develop [2]. Other factors, such as individual, social, environmental, and cultural factors also influence the prevalence of dental caries [3]. Visits to the dental practice have been associated with a lower prevalence of untreated dental caries as they are important to prevent tooth decay and to provide proper treatment [4]. Children with low socioeconomic support from their families have been found to present a greater prevalence of dental caries, probably due to the lack of dental visits and benefit from less preventive measures [5]. Therefore, evaluating the association between socioeconomic factors and oral health outcomes is very important. Previous studies have suggested that parents’ schooling and an increased number of residents in the household can be associated with dental caries. Investigating these factors is particularly important in developing countries, since the results may vary depending on regional differences [4,6,7]. Untreated dental caries affect the mastication function, speech, smile, and impact the quality of life of the child and family [1].

In children, dental caries may cause pain, discomfort, eating disorder, tooth loss and delayed speech. Furthermore, dental caries also affect children’s concentration in school and, at times, dental treatment expense may become a certain financial burden on the families, as it is very expensive in most countries. However, prevention is very simple and effective [2,8]. A physician or pediatrician can easily identify caries and habits of parents leading to caries in children and can counsel them for prevention and refer them to a specialist. Good oral hygiene measures, dietary counselling, and modification by decreasing the use of sugar and sticky food, as well as a healthy diet can help in preventing this disease in children. Nowadays, it is important to appraise all these factors for the pathways of dental caries prevention [1].

In order to assess the prevalence of dental caries in a population, it is important to consider international methods, amongst which, the most commonly known are the WHO (World Health Organization)—DMFT (Decay, Missing, Filled Tooth), the International Caries Detection and Assessment System (ICDAS) and the Caries Assessment Spectrum and Treatment (CAST). The ICDAS was first used in 2001 with the aim of creating a caries detection method that might be universal. Its major role was to allow clinicians, researchers, and epidemiologists to measure caries disease at different stages. Throughout the years, it was updated as ICDAD II for coronal and root surface, and for caries assessment associated with restorations and sealants (CARS). The ICDAS is now a two digit coding method; for caries, the method ranges from sound teeth (code 0), through enamel caries lesions (codes 1–3), to carious lesions in dentine (codes 4–6); for sealant and restoration, the method ranges from 0 to 8 as follows: 0 = Sound, 1 = Sealant, partial, 2 = Sealant, full, 3 = Tooth-colored restoration, 4 = Amalgam restoration, 5 = Stainless steel crown, 6 = Porcelain or gold or Porcelain-Fused-to-Metal (PFM) crown or veneer, 7 = Lost or broken restoration, 8 = Temporary restoration. Each surface is examined/coded, and when ICDAS is reported at tooth level, the worst condition is considered. More information about ICDAS is available from the website: http://www.icdas.org. Treatment needs are not considered within the method [9].

Recording and reporting data regarding oral health status can warrant the request for oral health resources and implementation of oral health programs. They can be used as a means to increase community awareness about oral health, promote community participation in preventive actions, and reorient oral health services from treatment to prevention [10]. WHO recommends to routinely collect epidemiological data in developed and developing countries for oral health surveillance. To the best of our knowledge, there are poor recent data regarding the oral health situation in Romania [11], as no comprehensive oral health surveys have been organized in the last five years. Some publications addressing this topic go back in 1987, when Petersen et al. [12] assessed the oral health situation of an industrial population in Romania. Baciu et al. in 2015 also published data regarding caries experience among Romanian school children [13].

In light of that, the objective of our pilot-study was to assess dental health status in 6 and 12-year old children in correlation with their family background, oral-health behavior and the intake of sweets.

## 2. Materials and Methods

A cross sectional dental health survey was conducted with the endorsement of the World Health Organization (WHO), the support of the Ministry of Health and also which complied with the General Data Protection Regulations 2018. The study methodology was approved by the relevant local (each school authority), regional (school inspectorate) and national authority (Ministry of Health from Romania—Approval No. 3411/05.04.2018). Informed consent for the children participating in the study was obtained from their parents/legal guardians prior to examination.

The study was conducted on 290 children from nine schools in the Transylvanian region of Romania ("Petru Rareș" Beclean National College; "Mihai Eminescu” Theoretical School Cluj-Napoca; Poieni Gymnasium School; "Andrei Șaguna" Barcani Secondary School; "Xántus János" Gymnasium School Miercurea Ciuc; Ruscova Technological School; "Vasile Netea" Technological School Deda, "Porolissum" Zalău Gymnasium School; Piscolt Gymnasium School): it comprised 149 children of 6 years of age and 141 children of 12 years of age. Children attending 0 and 6th grade of school were included in our study, their age of 6 years, respectively 12 years of age being the inclusion factor. Children that were below or above the selected age were not included in our study. In Romania, the schooling system starts from grade 0 (around the age of 6 for children: primary school (grades 0 to 4), followed by middle/secondary school (grades 5 to 8) and then either high school (grades 9 to 12) or vocational/ trade school (grades 9–13). Children who have graduated from high schools can achieve higher education by attending University. The selection of schools and classes that were examined was made in accordance with the WHO procedure for Oral Surveys in order to ensure that, even though the sample of population used was small, it was representative for the area population.

In Romania, there are schools that provide primary schooling, primary and middle schooling, middle and high/vocational-schooling, and schools that offer education from primary all the way until high-school graduation. Our study focused only on schools that had both 0 and 6th grade at the same premises. Schools were randomly selected from the official records of the Romanian Ministry for Education. First, for each school, we generated a random number between 1 and 4737 (i.e., the total number of schools in Romania) using the RANDBETWEEN function in MS Excel. Then, from each county, we selected the school that had the smallest random number. In Romanian schools, if a school has more than one class for each grade, these classes are differentiated using letters (e.g., classes 6A, 6B, 6C are different classes for the 6th grade). In the present study, if the school had more than one class with 12-year-old children, we selected different letters from different schools (i.e., in one school we selected the class 6A, and, in another school, we selected the class 6B). We used a similar approach for the selection of the classes with 6-year-old children. The nine schools that were included in our study were randomly selected from 1120 schools that fitted our inclusion criteria out of a total of 1182 schools in the area. In total, we were able to examine 149 6-year-old children out of 423 that frequented the nine schools and out of 42,973 children of the same age in the area. In addition, we examined 141 12-year-old children out of 398 that frequented the nine schools, from 42,149 children with the same age in the area. Table 1 shows enrollment size, sample distribution by region of residence, gender, and age.

**Figure 1:**
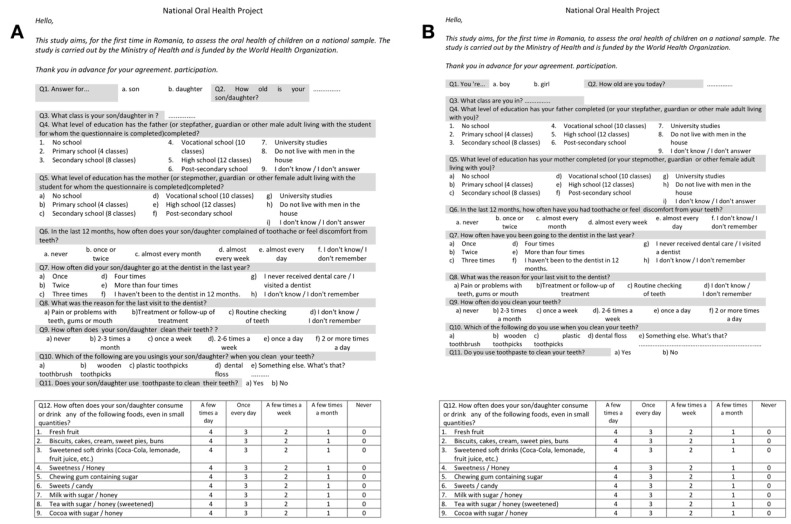
(**A**) questionnaire given for parents of 6-year-old children; (**B**) questionnaire given for 12-year-old children.

Prior to examination, informed consent forms as well as a questionnaire were distributed to the participants’ parents or legal guardian (Figure 1). The questionnaire was completed by the parents for the 6-year-old children, while the 12-year-old children completed it themselves and it included items referring to the child’s parental education, frequency and reason of visits to the dentist, dental care habits, and consumption of sweets.

The clinical survey was conducted by four experienced dentists, as well as four pediatric dentistry residents in pairs of 2. Before the examinations took place, the examiners underwent a calibration procedure following the WHO guidelines in order to have a uniform understanding and application of the International Caries Detection and Assessment System in both its original and modified version, as the intra-oral assessment of dental status was made using the modified ICDAS. The calibration ensured that each examiner could perform consistently to a specific standard. The calibration procedure was considered completed when a Cohen’s Kappa of 0.75 or higher was achieved for the group of examiners.

Children were clinically examined in their class at school following the WHO Oral Health Survey Methodology 5th Edition. The child was examined sitting, facing natural light, using cotton rolls to dry dental surfaces and a dental mirror only for indirect inspection, retraction of soft tissues or light re-directing purposes. Clinical findings were noted in a modified ICDAS chart for each participant.

A two-digit code was recorded, coding was done as follows: Restoration and Sealant Codes (0 = not sealed or restored; 1 = sealant, partial; 2 = sealant, full; 3 = tooth colored restauration; 4 = amalgam restauration; 5 = stainless steel crown; 6 = porcelain, gold, PFM crown or veneer; 7 = lost or broken restauration: 8 = temporary restauration); Caries Codes (0 = sound tooth surface; A = first visual change in enamel; B = distinct visual change in enamel; 3 = enamel breakdown, no dentine visible; 4 = dentinal shadow (not cavitated into dentine); 5 = distinct cavity with visible dentine; 6 = extensive distinct cavity with visible dentine); Missing Teeth (97 = extracted due to caries; 98 = missing for other reasons; 99 = unerupted).

Data were interpreted using descriptive statistics as well as a statistical software package Excel Data Analysis and Social Science Statistics (socscistatistics.com); mainly the *t*-test was used to find correlations. The statistically significant level was set as *p* < 0.05.

## 3. Results

In assessing the dental status of the enrolled children, the observations for each group of age were made according to the frequencies of the ICDAS scores considering all dental surfaces.

### 3.1. For 6-Year-Old Children, the Following Results Regarding ICDAS Score Were Registered

The most frequent ICDAS scores were “06” (extensive distinct cavity and visible dentine), 773 scores in male subjects and 422 scores in female subjects with a statistically significant difference between genders (*p* = 0.000218). By taking into consideration the place of residence for the subjects, a significant difference with regard to the presence of score “06” (extensive distinct cavity and visible dentine) was observed between children from the urban and rural areas in favor of the urban area, meaning the presence of the most serious score was more frequent in the rural area—630 scores opposed to 565 scores in the urban area; the first was related to 1236 affected surfaces compared with the second one which was related to 1007 affected surfaces (Table 2).

A statistically significant difference between genders at this age was found for the scores “20” and “30” (presence of sealants and tooth-colored restorations) with a higher number in females than in male subjects (*p* = 0.002339). Regarding the place of residence, only 1 score “30” with tooth-color restoration was found among the children living in the rural areas, whereas 42 were found in children living in the urban areas (*p* = 0.000076). In addition, no score “20” were found in children from the rural areas, while 17 scores “20” were present in children from the urban areas.

Both scores “03” (not sealed enamel breakdown, no dentine visible) and “05” (not sealed distinct cavity with visible dentine) had higher values for the children from the rural areas (145/57 and 191/114) with both differences being statistically significant (“03”—*p* = 0.00366; “05”—*p* = 0.005563). There were no statistically significant differences for the male/female scores for “03” and “04”.

The score “0A”—first visual change in enamel also presented differences in frequency. The frequency of the “0A” score was similar in male and female subjects (121, respectively 128); the frequency in children living in rural areas was increased (162) and a statistically significant difference in comparison with children from the urban area (89) (*p* = 0.001464) (Table 2) was apparent.

### 3.2. For 12-Year Old Children, the Following Results Regarding ICDAS Score Were Registered

The most frequent ICDAS scores registered were “0A” (first visual change in enamel), followed by “06” (tooth with extensive distinct cavity) and “99” (unerupted tooth). These findings were in accordance with the changing of dentition as many teeth were new; most untreated caries were found on first molars and there were many unerupted bicuspids, canines and second molars. For the “0A” score, no statistical differences were found between genders (*p* = 0.1481), nor between children having different residences (*p* = 0.20122) (Table 3).

A highly statistically significant difference was observed for the “06” score (tooth with extensive distinct cavity) between male and female subjects. While there was a 254 frequency for the “06” score in males, there was only a “99” frequency in females (*p* = 0.003141)—this indicates a better oral health in females. An important statistical difference was found for the same “06” score between children from urban and rural areas: the children from the urban areas displayed a 137 frequency in comparison with 221 in the rural areas (*p* = 0.019279). The “06” scores mostly referred to first permanent molars. They were often mistaken by parents during mixed dentition as deciduous teeth and go untreated for a long period of time due to a low frequency of visits to the dentist’s office.

Other significant differences were found for score “03” (*p* = 0.003614) with more cases for urban children, and, for score “04” (*p* = 0.0056), more cases were registered in the rural area, and, for score “05”, more cases registered for male (*p* = 0.001033). An important observation was that in the total of 141 children aged 12 years, no residual deciduous teeth were found.

### 3.3. Comparison of the ICDAS Scores between 6 and 12-Year-Old Children

**Figure 2:**
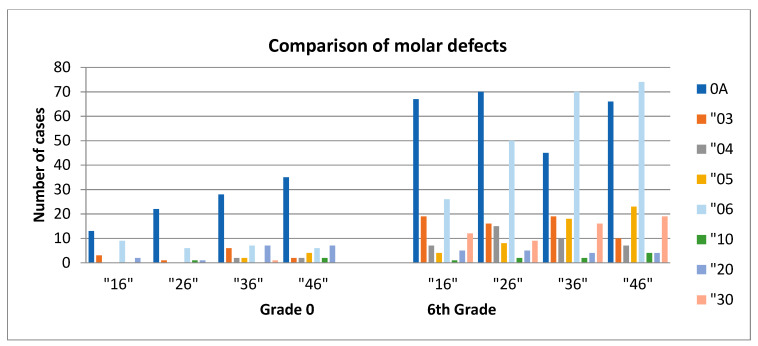
Comparison of ICDAS score on molar for 6 year and 12-year-old children.

A comparison between the ICDAS scores most commonly encountered between the children from the two age-based groups shows a statistically significant relation, as follows: the values for the “04”, “05”, and “06” scores from grade 0 were higher than those from the 6th grade (*p* < 0.01) and for “30” and “0A” the ratio was the other way around (*p* < 0.008); no significant differences were found for score 03. Due to the fact that the first molar is most likely to be affected by decay, we assessed the presence/ ICDAS code of the first molar in 6-year-old children and in 12-year-old children. In the 6-year-old group, we found the code “0A” for the first upper/lower and left/right molar in 98 children, whereas 248 “0A” codes were registered for 12-year-old children. The code “06“ was found in 28 6-year-old children and in 220 12-year-old children (Figure 2). No visible caries were recorded in 20 children (13.42%) in grade 0 and 24 children (17.02%) in the 6th grade.

### 3.4. Correlation between ICDAS Score and Family Background, Oral Health Practices and Diet

In order to evaluate possible factors that influence oral health for these children, we tried to find correlations between their family background, oral-health practices and the intake of sweets with the ICDAS scores.

#### 3.4.1. ICDAS Score Correlated with Family Background

For 6-year-old children, our tests indicated that the number of visits to the dentist in the last year, was correlated with the educational background of the parents. Parents who had graduated from a high-school or had a higher education demonstrated an increased number of visits for their children to the dentist in comparison with less qualified parents, although there were no statistical significant differences between the frequencies of visits and the educational background for either of the parents.

In 12-year old patients, we observed a lack of statistical correlation between the number of visits provided for their children to the dentist and their educational background, even though most fathers were high-school graduates (double the frequency of all other qualifications).

An important percentage of children/parents did not answer the question about why they visited the dentist. For grade 0, 96 (64.23%) of the parents and for 6th grade, 85 (60.28%) of the children specified the reason for visiting the dentist. On the other hand, the reason for visits to the dentist depended on the level of qualification of the parents/tutors in the sense that the number of routine checks were higher for those with tertiary education (Table 4).

Comparing the reason for a child’s visit to the dentist were routine check-up and/or pain. In the frequency of different ICDAS scores, there were statistically significant differences, the frequency of ICDAS scores being systematically less for those who attended a regular check-up for “03”, “04”, and “05” scores in both grades. For 12-year-old children, there was also a statistically significant difference for the “06” score and a lower frequency for children with a regular check-up than for those with the pain reason for a dental visit (*p* = 0.003186).

The high percentage of those who either had not been to the dentist in the last 12 months, or never had been, decreased with the rise of the education level of parents/tutors. For example, for those with tertiary education, the answer to had “never” been was less than 7%, for both grades, less than 20% for high school and reaching over 50–60% for primary or less education. In addition, in both grades, the highest percentage of a single visit in the last year was found up to 40%, when the parent’s education level was only primary school.

#### 3.4.2. ICDAS Score Correlated with Oral Health Practices

**Figure 3:**
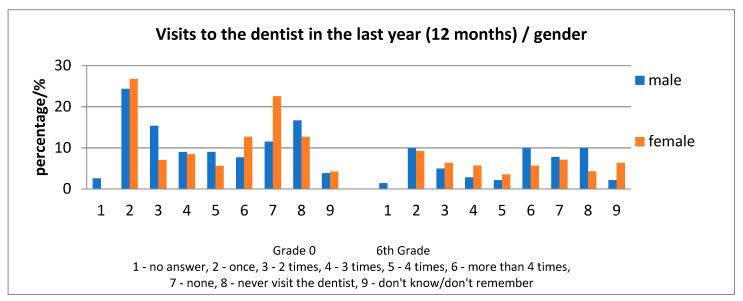
Differences of the number of visits in the dental office for the last year, according to gender.

**Figure 4:**
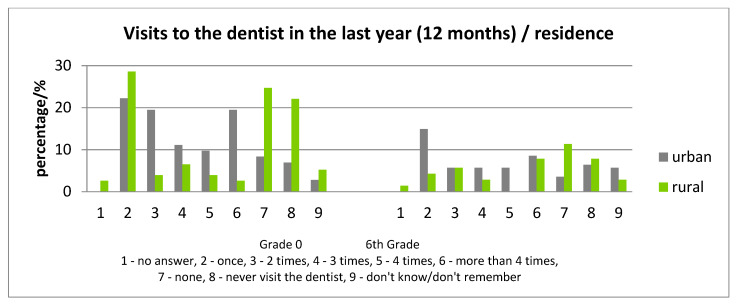
Differences of the number of visits in the dental office for the last year, according to residence.

In 6-year old children, the highest frequency of an answer in regard to the question concerning the number of visits to the dentist during the last year, was one visit, followed by 0 visits or never having provided a visit for the child at the dentist (Figure 3). In 12-year-old children, the situation was found to be slightly different as the highest frequency of answer regarding the question concerning the number of visits was: one visit, followed by more than four visits in a year, then 0 visits or never (Figure 4).

Analyzing the frequency of visits to the dentist in correlation with the residence of the child, children from rural areas evidenced less visits to the dentist (Figure 3). Those who never visited a dentist represented 22.08% (grade 0) and 17.74% (6th grade) in rural areas compared with 8.33% (grade 0) and 11.39% (6th grade) in the urban area and, for the “no visit in the last year”, the values are 24.68% (grade 0) and 25.81% (6th grade) in the rural area, and 6.33% (6th grade) and 6.94% (grade 0) in the urban area. Assessment of the ICDAS scores in children “who never visited a dentist” is indicated in Table 5.

**Figure 5:**
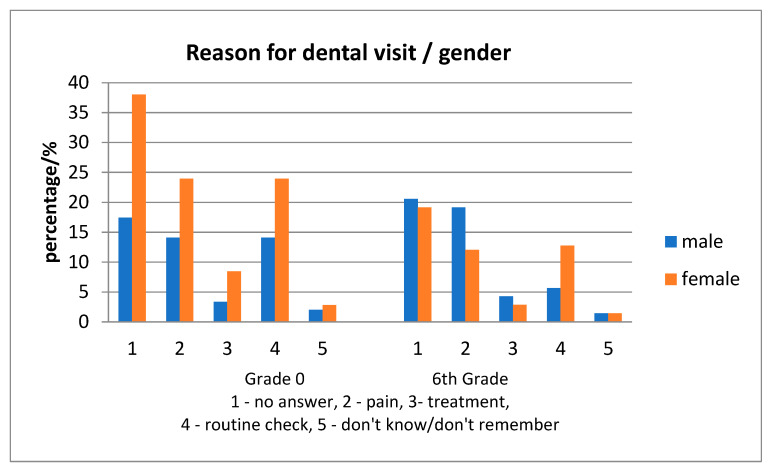
Differences in the reason for visits in the dental office for the last year, according to gender.

**Figure 6:**
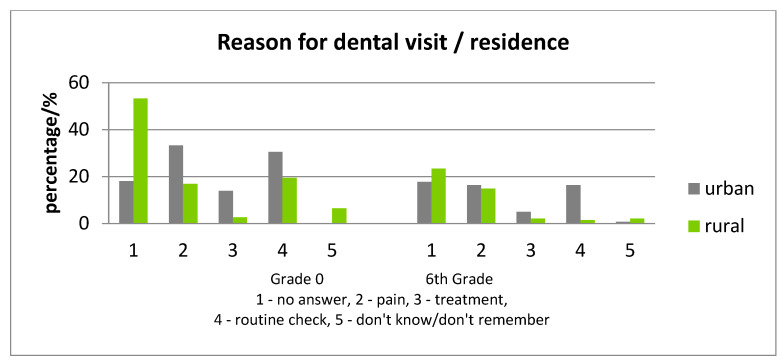
Differences in the reason for visits to the dental practice for the last year, according to residence.

No statistically significant differences were found among the reasons for the visit at the dentist, between gender (Figure 5). In all children, the reason for the visit to the dentist was equally the presence of dental pain or a regular check-up, while the frequency of a regular check-up as a motive was mostly found in urban children (Figure 6).

Regarding dental hygiene, the use of the toothbrush and toothpaste was evident over 90–95% for each grade, regardless of gender and location. There were only a few cases (less than four in each age group), where the children used an electric toothbrush or toothpicks and dental floss. The teeth cleaning frequency is shown in Table 6.

Most children cleaned their teeth one or more times per day. Significant differences appeared between urban and rural areas in both age-groups, rural areas having a lower percentage of children cleaning their teeth twice or more times per day. Regarding gender, males from the 6th grade had a lower percentage for once per day cleaning, but a higher percentage for two or more times per day. There were statistically significant differences between urban and rural areas in the 6th grade, the values for urban areas being less than those for rural areas for score “04” (*p* = 0.016482), “05” (*p* = 0.039127), “06” (*p* = 0.010785) when one considers the two or multiple times teeth cleaning. For the 6th grade, the “05” score in males had higher values than for females, a difference significantly statistical (*p* = 0.00934) at a once per day cleaning habit.

#### 3.4.3. ICDAS Score Correlated with Eating Habits

**Figure 7:**
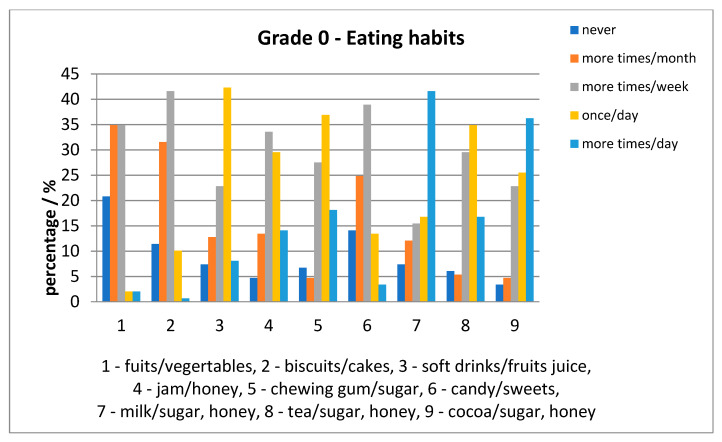
Eating habits in 6-year-old children.

**Figure 8:**
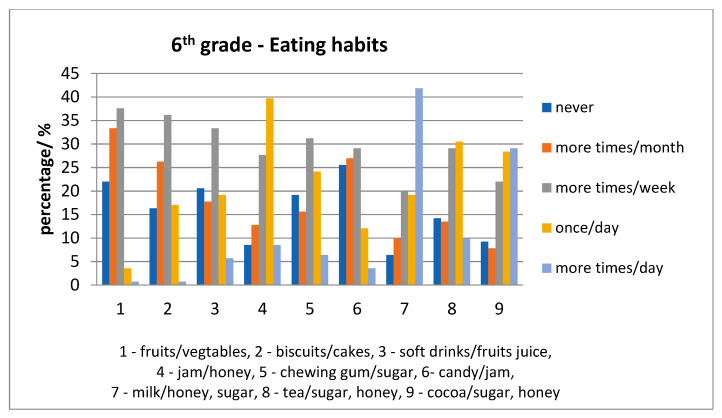
Eating habits in 12-year-old children.

Relative to eating habits, for each age category, fruits/vegetables were consumed less often than milk with sugar/honey. In addition, other foods were consumed once a day (e.g., milk, tea and cocoa with sweetener) were consumed repeatedly; the daily situation is presented in Figure 7 and Figure 8.

Only 77 (out of 149) of the 6-year-old children and 43 (out of 141) of the 12-year-old children consumed all nine foods questioned; the ICDAS scores are indicated in Table 7.

**Figure 9:**
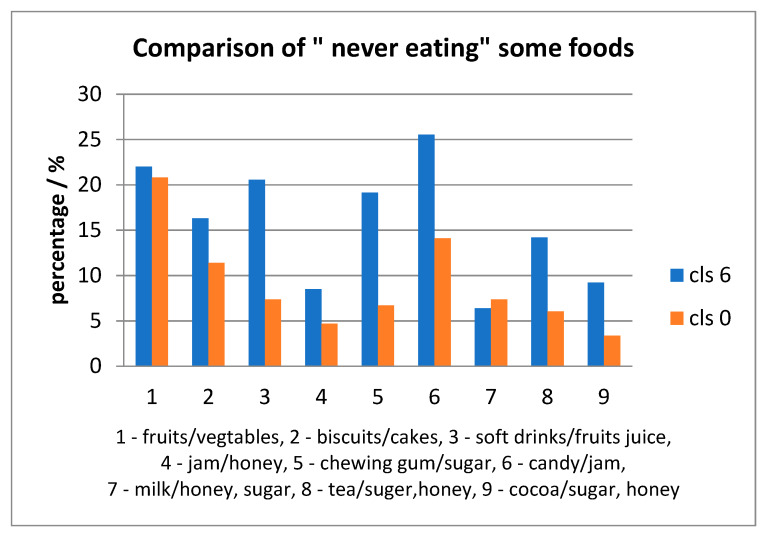
Comparison of “never eating “foods between 6- and 12-year children.

For each item taken in the survey, there were children who declared: to have “never” consumed it. The percentages were high for fruits/vegetables for both age-groups; also, the percentages for other items were higher for the 6th grade rather than grade 0, except milk with sugar/honey, presented in Figure 9. The eating habits in both age groups were compared to the “03”, “04”, “05”, “06”, “0A” ICDAS score of each child. There were statistically significant differences for: jam/honey (*p* = 0.001149) and chewing gum with sugar (*p* = 0.03749) consumed several times daily with higher scores for grade 0 than 6th grade. Another statistically significant difference was for biscuits/cakes/sweet pies (*p* = 0.011009), soft drinks/fruits juice (*p* = 0.025494), jam/honey (*p* = 0.0223456), chewing gum with sugar (*p* = 0.02287) consumed once a day with higher values for 6-year old than 12-year old children.

## 4. Discussion

In spite of the significant reduction in caries prevalence in many parts of the world, dental caries remains a major public health problem affecting people of all ages [14]. The process of choosing the best model to evaluate an oral disease needs to represent a balance between simplicity and accuracy. This is particularly true for caries disease in childhood, as cooperation with children has proved to be more difficult. Caries assessment methods have the goal of evaluating and recording consistent and standardized data of tooth condition, providing information that can be used for clinical, research, and epidemiological purposes [9].

The present study reported relevant data regarding the oral health status of 6 and 12-year-old children from nine schools situated in the Transylvania and Central Region of Romania in correlation with their family background, oral-health behavior and intake of sweets. Our results indicated that caries codes (“0A”, “03”, “05”, “06”) recorded for 6-year-old children were more prevalent in the rural areas, and in male patients (“0A”, “06”). Restoration and sealant code “30” were more prevalent in the urban areas in female children. This indicates that, in the rural areas, dental cavities are mostly left untreated, evolving to “06” code. In the urban areas, dental caries were treated especially in female children, as females are more preoccupied by health and aspect issues and more cooperative.

Recorded caries code for 12-year-old children demonstrated no statistically significant differences between rural/urban, male/female children for code “0A”; “03” code was prevalent in urban areas, whereas, “04” and “05” codes were higher in the rural areas for male children. The highest recorded code 06 was more prevalent in the rural areas for male children. In addition, 12-year-old children from rural areas presented more advanced caries lesions compared to children from the urban areas. Data recorded for 12-year-old children demonstrated that the trend of untreated dental cavities at 6 years was transmitted in 12-year-old children in the rural areas. Recorded data indicated that Romania has not yet met the WHO targets for dental caries for 2020; 50% of 5–6-year-old children were caries free [15].

When comparing caries codes from 6 to 12-year-old children, initial lesions, encoded with 0A were more prevalent in 12-year-old children. This correlated with the fact that in 6-year-old children caries lesions were in a more advanced stage (score “04”,”05”, “06”). Restoration and sealant codes (“30”) were more prevalent in 12-year-old children, as there was a trend to leave caries lesions untreated for temporary dentition. Due to the fact that the first molar is most likely to be affected by decay, we assessed the presence/ICDAS code of the first molar in 6-year-old children and in 12-year-old children. As the first molar shows at 6-years, caries codes were prevalent in 12-year-old children.

Comparing oral health status with the family background demonstrated that there was a strong correlation in 6-year-old children, as parents with a higher education visited the dentist more often with their children. For 12-year-old children, there was a lack of correlation between the parent’s background and the ICDAS score. This might be due to the fact, that at 12 years, children were more independent. The reason for a dental visit was also correlated with the parent’s background; parents with a higher education took their children for more routine checkups. For those children who reported a higher number of dental visits, the prevalence of the registered ICDAS scores was lover in 12-year-old children. This underlined the importance of primary and secondary prevention of caries. Our results indicated that the number of those children who had not attended a dentist in the last 12 months decreased according to the higher education level of the parents. Most of the 6-year-old children went once per year to the dentist, whereas most of the 12-year-old children had more than four visits in a year. The increasing number of dental visits in 12-year-old children indicated a higher treatment need in this group, due to the lack of proper education in 6-year-old children. The number of dental visits was higher in the urban population, which correlated with better education strategies both for parents and children. In the urban area, the main reason for dental visits was the routine checkup.

Comparing oral health status with oral hygiene, most 6 and 12-year-old children brushed once a day. Children from the urban areas brushed more than once per day. If we refer only to those 12-year-old children who brushed twice or more than twice per day, our results demonstrated a lower prevalence of the ICDAS scores (“04”, “05”, “06”) in the urban area.

Recording of eating habits indicated that fruits/vegetables were not consumed daily. All other foods assessed in the questionnaire that we compiled, were consumed once a day. Eating habits in both age groups were associated with statistically significant differences of “03”, “04”, “05”, “06”, “0A” ICDAS score of each child for jam/honey and chewing gum with sugar consumed several times daily with higher scores for grade 0 than 6th grade and for biscuits/cakes/sweet pies, soft drinks/fruits juice, jam/honey, chewing gum with sugar, consumed once a day with higher values for grade 0 than 6th grade. Our results indicated a decrease in the consumption frequency of chewing gum with sugar, biscuits/cakes/sweet pies, soft drinks/fruits juice, jam/honey from 6 to 12-year children. This indicated a better cooperation with 12-year-old children.

The results of our study demonstrated that ICDAS scores differences for rural/urban areas, for 6 and 12-year-old children were associated with the family background, oral hygiene (number of visits to the dental practice, oral hygiene) and eating habits. These data will lay the foundation of implementing community-based oral health promotion interventions to reduce the burden of dental caries in the Romanian population. These strategies will include primary prevention methods addressing resistance to disease progress and secondary prevention—aiming to stop the progress of dental disease. Primary prevention methods could include: the use of fluorides, sugar substitutes, mechanical barriers-pit and fissure sealants, and health education in schools and kindergartens [16]. Secondary prevention would aim to have an early diagnosis and prompt treatment to reduce lesion complication.

The main limitation of this survey lies in the calibration process itself. In theory, calibration is based on the assumption that only true scores are recorded by a gold standard (instrument or examiner), which is theoretically 100% error-free. In clinical oral settings, the scores are generated by a benchmark scorer, usually an experienced examiner who is assumed to be error-free or nearly so, but, of course, some misclassification errors are expected [9]. However, before starting the clinical examinations, all examiners underwent this calibration procedure and it was considered completed when a Cohen’s Kappa of at least 0.75 was achieved by all examiners. A second limitation of our study that should be considered, is the small sample of examined children. Our methodology complied with the WHO Pathfinder methodology which allows the examination of only a small sample of the population. However, the randomization process ensured that despite the size, the sample was representative of the studied population; in our case, the population of 6-year old children and 12-year-old children from Transylvania and the central region of Romania. Another limitation consists of the examination process of children, as examinations were done on school premises, in classrooms, with natural and electric light and not in a dental chair with adequate illumination for intra-oral examination and the possibility to air-dry the examination field. However, all examiners were calibrated for assessment in the same conditions prior to the beginning of the survey.

On the other hand, one of the strengths of the present survey is the wide sample included. To the authors’ knowledge, no other recent study has included complete and complex calibration and data analysis on a population selected so thoroughly, providing more credit to the validity of the findings [9]. In addition, it is the first study where the prevalence of dental decay was also correlated with socio-economic factors in the family, frequency of visits to the dental practice and oral-hygiene and dietary habits. Another strength is that this survey investigated many risk factors reportedly associated with caries, such as dietary habits, oral hygiene practices, dental visits, and the parents’ level of education. Such information provides clarification on the potential risk factors of caries among this population, and therefore, it can be used as a starter point for future studies to investigate in depth, the effects of various variables on caries experience among Transylvanian schoolchildren. In addition, despite the sample size, the fact that the study was carried out in both populous and multicultural urban centers as well as less populated rural areas, the generalizability of the results to all Romanian schoolchildren is less questionable. However, further studies that would cover different regions of the country are required [14].

## 5. Conclusions

Our findings revealed that the examined children presented a high prevalence of dental caries and a large amount of untreated carious teeth. In addition, it was observed that their presence in the dental practice in the past year was correlated with the parents’ level of education. Given the high prevalence of dental caries and extensive treatment needed, more studies that investigate, in depth, the associated risk factors are highly recommended in the area and using a larger population. A National Health Program dedicated to prevention and early detection and treatment of dental caries in children should be implemented. In addition, within the educational curriculum, health and hygiene habits should be emphasized.

## Figures and Tables

**Table 1:** Sample description according to gender, age, and residence distribution.

Population Distribution	6-Year-Old Children (Grade 0)		12-Year-Old Children (6th Grade)		Total
	Female	Male	Female	Male	
Urban	38	34	44	35	151
Rural	33	44	24	38	139
Total	71	78	68	73	290

**Table 2:** Most frequent ICDAS scores for the 6-year-old group (percentage of total cases for male and female respectively, urban to rural; percentages calculated to the total numbers of cases for each category).

Grade 0				
ICDAS Score	Male	Female	Urban	Rural
03	101 (7.84%)	101 (10.62%)	57 (5.68%)	145 (11.72%)
04	116 (9%)	89 (9.36%)	96 (9.56%)	109 (8.81%)
05	156 (12.1%)	142 (14.93%)	114 (11.16%)	184 (14.87%)
06	773 (59.97%)	422 (44.37%)	565 (56.27%)	630 (50.93%)
20	5 (0.39%)	12 (1.26%)	17 (1.69%)	0
30	8 (0.62%)	35 (3.68%)	42 (4.18%)	1 (0.08%)
0A	121 (9.39%)	128 (13.46%)	87 (8.76%)	162 (13.1%)
Number of Missing Teeth	93	82	92	83
97	19 (20.43%)	31 (37.8%)	29 (31.52%)	21 (25.39%)
98	6 (5.38%)	1 (1.22%)	1 (1.09%)	6 (7.23%)
99	68 (74.19%)	50 (60.98%)	62 (67.39%)	56 (67.47%)

**Table 3:** Most frequent ICDAS scores for the 12-year-old group (percentage of total cases for male and female respectively, percentages calculated to the total numbers of cases for each category).

6th Grade				
ICDAS	Male	Female	Urban	Rural
03	86 (10.1%)	75 (10.8%)	108 (13.9%)	53 (6.52%)
04	64 (7.36%)	52 (7.69%)	40 (5.15%)	78 (9.59%)
05	77 (8.91%)	26 (3.42%)	37 (4.76%)	66 (8.12%)
06	254 (29%)	99 (14.73%)	137 (17.6%)	221 (27.18%)
10	9 (1%)	4 (0.66%)	13 (1.67%)	0
20	27 (1.66%)	16 (1.54%)	40 (5.15%)	3 (0.37%)
30	52 (5.58%)	50 (8.38%)	63 (8.11%)	39 (4.8%)
0A	311 (34.33%)	339 (50.45%)	308 (39.6%)	346 (42.6%)
Number of Missing Teeth	115	123	145	96
97	12 (17.9%)	10 (15.7%)	3 (2.76%)	19 (19.8%)
98	0	3 (5.88%)	2 (1.38%)	1 (1.04%)
99	103 (82.1%)	110 (78.4%)	139 (95.9%)	76 (79.2%)

**Table 4:** Correlation between parents’ education and reason for a dental visit in 12 and 6-year-old children.

6th Grade					Grade 0			
Mother/Tutor	No Answer	Pain	Treatment	Routine Check	No Answer	Pain	Treatment	Routine Check
Primary Education	8 (100%)				5 (62.5%)	2 (25%)		1 (12.5%)
Secondary School	11 (55%)	6 (30%)		1 (5%)	12 (69%)	4 (20%)		1 (5%)
Vocational School	12 (40%)	13 (43.33%)	4 (13.33%)	1 (3.33%)	6 (30%)	8 (40%)		5 (25%)
High School	11 (28.21%)	13 (33.33%)	3 (7.69%)	10 (25.64%)	12 (31.57%)	12 (31.57%)	5 (13.15%)	8 (21.06%)
Tertiary Education	10 (30.3%)	7 (21.21%)	3 (9.09%)	13 (39.39%)	7 (14.53%)	12 (25%)	6 (12.5%)	22 (45.83%)
Father/Tutor								
Primary School	5 (71.42%)	1 (14.28%)	1 (14.28%)		4 (57.14%)	1 (14.28%)		1 (14.28%)
Secondary School	8 (47.05%)	8 (47.05%)	1 (5.89%)		9 (47.62%)	5 (23.81%)		4 (19.04%)
Vocational School	12 (54.54%)	6 (27.28%)	1 (4.54%)	3 (13.63%)	7 (24.13%)	13 (44.82%)	4 (13.79%)	4 (13.79%)
High School	16 (29.62%)	20 (37.03%)	6 (11.11)	9 (16.67%)	18 (40%)	9 (20%)	3 (6.67%)	14 (21.05%)
Tertiary Education	8 (25.92%)	5 (18.51%)	2 (7.41%)	13 (48.14%)	2 (6.67%)	9 (30%)	4 (13.34%)	15 (50%%)

The differences up to 100% are given by “don’t know/don’t answer”.

**Table 5:** Contribution to ICDAS score of the children who “never visited a dentist”.

	03 (%)	04 (%)	05 (%)	06 (%)	20 (%)	30 (%)	0A (%)
Grade 0	32 (15.84%)	22 (10.73%)	39 (13.09%)	183 (15.31%)	1 (2.33%)	48 (19.28%)	32 (15.84%)
6th Grade	32 (19.88%)	12 (10.17%)	16 (15.53%)	154 (38.83%)	6 (5.88%)	64 (11.31%)	32 (19.88%)

**Table 6:** Teeth cleaning frequencies.

Grade 0						6th Grade				
		Female	Male	Urban	Rural		Female	Male	Urban	Rural
No Answer	2 (1.34%)	0	2 (1.34%)	0	2 (1.34%)	5 (3.55%)	4 (2.84%)	1 (0.71%)	4 (2.84%%)	1 (0.71%)
Never	5 (3.36%)	2 (1.34%)	3 (2.01%)	0	5 (3.36%)	2 (1.42	1 (0.71%)	1 (0.71%)	0	2 (1.42%)
2–3 Times/Month	4 (2.68%)	0	4 (2.68%)	1 (0.67%)	3 (2.01%)	7 (4.96	1 (0.71%)	6 (4.26%)	1 (0.71%)	6 (4.26%)
Once/Week	10 (6.71%)	5 (3.36%)	5 (3.36%)	2 (1.34%)	8 (4.7%)	5 (3.55	1 (0.71%)	4 (2.84%)	3 (2.13%)	2 (1.42%)
2–6 Times/Week	11 (7.38%)	6 (4.03%)	5 (3.36%)	5 (3.36%)	6 (4.03%)	15 (10.64	7 (4.96%)	8 (5.67%)	6 (4.26%)	9 (6.38%)
Once/Day	67 (44.97%)	32 (21.48%)	35 (23.49%)	33 (22.15%)	34 (22.15%)	42 (29.79	18 (12.77%)	24 (17.02%)	26 (18.44%)	16 (11.35%)
2 or More Times/Day	50 (33.56%)	26 (17.45%)	24 (16.11%)	30 (20.81%)	20 (13.42%)	65 (46.1%)	36 (25.53%)	28 (19.86%)	39 (27.66%)	26 (18.44%)

**Table 7:** ICDAS score registered for 6 and 12-year-old children consuming all nine foods listed.

Percentage of the Total Scores for Each Item	03	04	05	06	20	30	0A
Grade 0	102 (50.51%)	112 (58%)	147 (49.49%)	431 (36.11%)	15 (88.24%)	29 (67.44%)	130 (52.2%)
6th Grade	31 (19.25%)	21 (17.8%)	12 (11.65%)	81 (22.63%)	34 (79.07%)	39 (38.24%)	179 (27.3%)

## References

[1] Mathur V.P., Dhillon J.K. (2018). Dental caries: A disease which needs attention. Indian J. Pediatr..

[2] Bramantoro T., Setijanto R.D., Palupi R., Aghazy A.Z., Irmalia W.R. (2019). Dental caries and associated factors among primary school children in metropolitan city with the largest Javanese race population: A crosssectional study. Contemp. Clin. Dent..

[3] Prabakar J., Arumugham I.M., Sri Sakthi D., Kumar R.P., Leelavathi L. (2020). Prevalence and comparison of dental caries experience among 5 to 12 year old school children of Chandigarh using dft/DMFT and SiC index: A cross-sectional study. J. Fam. Med. Prim. Care.

[4] da Dutra L.C., Neves E.T.B., de Lima L.C.M., Gomes M.C., Forte F.D.S., Paiva S.M., de Abreu M.H.N.G., Ferreira F.M., Granville-Garcia A.F. (2020). Degree of family cohesion and social class are associated with the number of cavitated dental caries in adolescents. Braz. Oral Res..

[5] Fontanini H., Marshman Z., Vettore M. (2015). Social support and social network as intermediary social determinants of dental caries in adolescents. Community Dent. Oral Epidemiol..

[6] Saldunaite K., Bendoraitiene E.A., Slabsinskiene E., Vasiliauskiene I., Andruskeviciene V., Zubiene J. (2014). The role of parental education and socioeconomic status in dental caries prevention among Lithuanian children. Medicina (Kaunas).

[7] de Vazquez F.L., Cortellazzi K.L., Kaieda A.K., Bulgareli J.V., Mialhe F.L., Ambrosano G.M.B., da Silva Tagliaferro E.P., Guerra L.M., de Castro Meneghim M., Pereira A.C. (2015). Individual and contextual factors related to dental caries in underprivileged Brazilian adolescents. BMC Oral Health.

[8] Zhang S., Liu J., Lo E.C.M., Chu C.-H. (2014). Dental caries status of Bulang preschool children in Southwest China. BMC Oral Health.

[9] Campus G., Cocco F., Ottolenghi L., Cagetti M.G. (2019). Comparison of ICDAS, CAST, Nyvad’s criteria, and WHO-DMFT for caries detection in a sample of italian schoolchildren. Int. J. Environ. Res. Public Health.

[10] Petersen P.E., Baez R.J. (2013). Oral Health Survey: Basic Methods.

[11] Petersen P.E. (2009). Global policy for improvement of oral health in the 21st century—Implications to oral health research of World Health Assembly 2007, World Health Organization. Community Dent. Oral Epidemiol..

[12] Petersen P.E., Tanase M. (1997). Oral health status of an industrial population in Romania. Int. Dent. J..

[13] Baciu D., Danila I., Balcos C., Gallagher J.E., Bernabe E. (2015). Caries experience among Romanian schoolchildren: Prevalence and trends 1992–2011. Community Dent. Health.

[14] Al-Akwa A.A., Al-Maweri S.A. (2018). Dental caries prevalence and its association with fluoride level in drinking water in Sana’a, Yemen. Eur. J. Dent..

[15] Federation Dentaire Internationale (1982). Global goals for oral health in the year 2000. Int. Dent. J..

[16] de Silva A.M., Hegde S., Akudo Nwagbara B., Calache H., Gussy M.G., Nasser M., Morrice H.R., Riggs E., Leong P.M., Meyenn L.K. (2016). Community-based population-level interventions for promoting child oral health. Cochrane Database Syst. Rev..

